# 3D Culture of MSCs on a Gelatin Microsphere in a Dynamic Culture System Enhances Chondrogenesis

**DOI:** 10.3390/ijms21082688

**Published:** 2020-04-13

**Authors:** Shamsul Sulaiman, Shiplu Roy Chowdhury, Mh Busra Fauzi, Rizal Abdul Rani, Nor Hamdan Mohamad Yahaya, Yasuhiko Tabata, Yosuke Hiraoka, Ruszymah Binti Haji Idrus, Ng Min Hwei

**Affiliations:** 1 Tissue Engineering Centre, Universiti Kebangsaan Malaysia, Kuala Lumpur 56000, Malaysia; sshamsul@ppukm.ukm.edu.my (S.S.); shiplu56@gmail.com (S.R.C.); fauzibusra@ukm.edu.my (M.B.F.); ruszyidrus@gmail.com (R.B.H.I.); 2 Department of Orthopedic & Traumatology, Universiti Kebangsaan Malaysia, Kuala Lumpur 56000, Malaysia; rizal@ppukm.ukm.edu.my (R.A.R.); nhmycj@gmail.com (N.H.M.Y.); 3 Department of Biomaterials, Institute for Frontier Medical Sciences, Kyoto University, 53 Kawara-cho Shogoin, Sakyo-ku Kyoto 606-8507, Japan; yasuhiko@frontier.kyoto-u.ac.jp; 4 Biomaterial Group, R&D Center, Nitta Gelatin Inc, 2-22, Futamata, Yao City, Osaka 581-0024, Japan; yo-hiraoka@nitta-gelatin.co.jp; 5 Department of Physiology, Universiti Kebangsaan Malaysia, Kuala Lumpur 56000, Malaysia

**Keywords:** gelatin microsphere, microcarrier, cartilage, osteoarthritis, tissue engineering

## Abstract

Recent advancement in cartilage tissue engineering has explored the potential of 3D culture to mimic the in vivo environment of human cartilaginous tissue. Three-dimensional culture using microspheres was described to play a role in driving the differentiation of mesenchymal stem cells to chondrocyte lineage. However, factors such as mechanical agitation on cell chondrogenesis during culture on the microspheres has yet to be elucidated. In this study, we compared the 2D and 3D culture of bone-marrow-derived mesenchymal stem cells (BMSCs) on gelatin microspheres (GMs) in terms of MSC stemness properties, immune-phenotype, multilineage differentiation properties, and proliferation rate. Then, to study the effect of mechanical agitation on chondrogenic differentiation in 3D culture, we cultured BMSCs on GM (BMSCs-GM) in either static or dynamic bioreactor system with two different mediums, i.e., F12: DMEM (1:1) + 10% FBS (FD) and chondrogenic induction medium (CIM). Our results show that BMSCs attached to the GM surface and remained viable in 3D culture. BMSCs-GM proliferated faster and displayed higher stemness properties than BMSCs on a tissue culture plate (BMSCs-TCP). GMs also enhanced the efficiency of in-vitro chondrogenesis of BMSCs, especially in a dynamic culture with higher cell proliferation, RNA expression, and protein expression compared to that in a static culture. To conclude, our results indicate that the 3D culture of BMSCs on gelatin microsphere was superior to 2D culture on a standard tissue culture plate. Furthermore, culturing BMSCs on GM in dynamic culture conditions enhanced their chondrogenic differentiation.

## 1. Introduction

Tissue Engineering and regenerative medicine have been considered as a potential alternative to conventional treatment for cartilage diseases [1]. Typically, the application of the technology demonstrates the regeneration of the injured tissue, returning its functional state [1]. Stem cells, the origin of every other cell, have been the epitome of this therapeutic miracle for tissue regeneration [1]. Numerous preclinical studies have shown stem cells’ efficacy on reviving damaged cartilage tissues to its functional state [2,3,4]. Besides, more than 200 clinical trials have been conducted to treat cartilage damage using stem cells to determine the safety, efficacy, and long-term feasibility in patients [5], some of these were proven effective, while some were not [5].

Mesenchymal stem cells (MSCs) were frequently regarded as the cell of choice for cartilage disease due to its characteristics, such as multipotency, low immunogenicity, and safety [6]. Based on the available data in the literature, a large dosage of MSCs (10–100 million cells) is required in order to demonstrate clinical benefits for a patient [7]. Various clinical studies have utilized monolayer 2D cell stack culture chambers in order to meet the required dose of cells [8,9,10], which require an extended period of in vitro expansion. Numerous reports have shown the loss of their stemness properties, including mitotic ability and colony-forming unit efficiency in long-term in vitro expansion [11,12,13,14]. On top of that, it was also reported that MSCs would tend to lose their chondrogenic potential with culturing time [11]. This claim could explain the failures of several clinical studies beforehand. Hence, there is a necessity to fill this gap in order to preserve its functionality and regenerative capabilities.

The use of a three-dimensional (3D) cell culture system has recently demonstrated a significant impact on improving cell proliferation while preserving cell phenotype and biological cues [15]. The 3D culture system facilitates cell–cell and cell–extracellular matrix (ECM) interaction, permitting cells to adapt to their native morphology and subsequently influence the signaling activity. A few approaches were introduced to create a 3D microenvironment by fabricating various scaffolds (hydroxyapatite (HA), tricalcium phosphate-hydroxyapatite (TCP-HA) and gelatin) using natural and synthetic biomaterials [16,17,18,19]. Extensive studies on various scaffolds have been effectively employed with multiple cell types and yielded promising outcomes [18,20,21,22].

Microspheres are widely known for their usage in drug delivery and biological agents [23]. Besides, commercially available microspheres, such as Cytodex 1, CultiSpher S, and SphereCol, were also utilized for the in vitro expansion of cells in a bioreactor [22,24,25]. The microsphere provided a 3D culture environment for cell expansion and was found to shorten the expansion process as it provides a higher surface to volume ratio [25,26,27] for cell proliferation. Gelatin microspheres (GMs) are biocompatible and have been shown to promote adhesion and growth of various cell types, including MSC [28,29,30,31]. While the goal of utilizing a 3D culture system is to generate a higher amount of MSCs in a shorter period, improving their chondrogenic differentiation is also important. In this regard, careful selection of a specific culture medium would be paramount. Due to the robust native microenvironment of cartilage tissue, it was speculated that mechanical stimuli could also influence the behavior of the native cells. Previous studies have supported the notion and demonstrated that mechanical stimuli could improve collagen matrix formation and increased expression of collagen type II [14,32,33]. With these in mind, the combination of 3D culture system (to improve the proliferation of MSC), and mechanical agitation (to improve chondrogenic differentiation) would be an ideal manufacturing design for MSC-based therapies for cartilage damage.

Thus, our current work aims to explore the potential effect of the 3D culture system in a dynamic culture environment on GMs cultured with bone-marrow-derived MSC (BMSCs). In this study, we established a 3D culture system with BioLevitator^TM^ as the hybrid bioreactor for a microcarrier-based culture. The BioLevitator^TM^ provides a full mixing environment via the blades at the bottom of the culture tube with tunable parameters that produce dynamic culture conditions (mechanical agitation). To accomplish this, we first investigate the capability of BMSCs cultured in a 2D and 3D system. The morphology, proliferation, immunophenotype activity, preservation of stemness properties, and the interaction of BMSCs on fabricated GMs were analyzed. The effect of two types of culture environment (static and dynamic) and culture medium; F12: DMEM + 10% FBS (FD) and chondrocytes induction medium (CIM) on BMSCs chondrogenic differentiation were also studied.

## 2. Results

### 2.1. Characterization of Gelatin Microspheres

**Figure 1:**
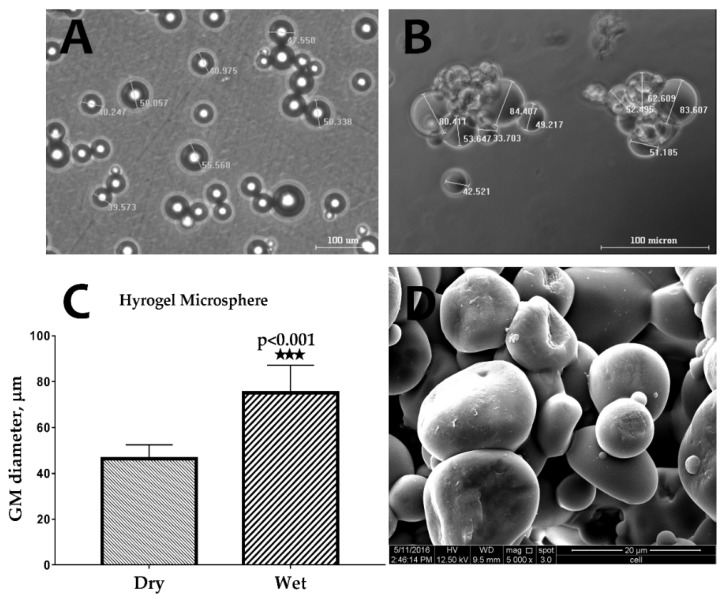
Optical microscope images of gelatin microspheres (GMs) in (**A**) dry and (**B**) wet conditions. (**C**) The swelling measurement of hydrogel microsphere (*n* = 6) (*** *p* < 0.001), and (**D**) an scanning electron microscope (SEM) image of GMs showing the sphericity and smooth surface of the GMs.

The images captured using an optical microscope of the GM in dry and wet conditions show significant swelling of the GMs after their immersion in PBS (Figure 1A,B). As shown in Figure 1C, the size of the GMs in the wet condition (74.3 ± 6.84 µm) was approximately 1.7 times higher than in the dry condition (43.90 ± 2.44 µm). Under scanning electron microscope (SEM) analysis, the GM demonstrated a spherical shape, and the surface was found to be smooth (Figure 1D).

### 2.2. Morphology of BMSCs on a Gelatin Microsphere

**Figure 2:**
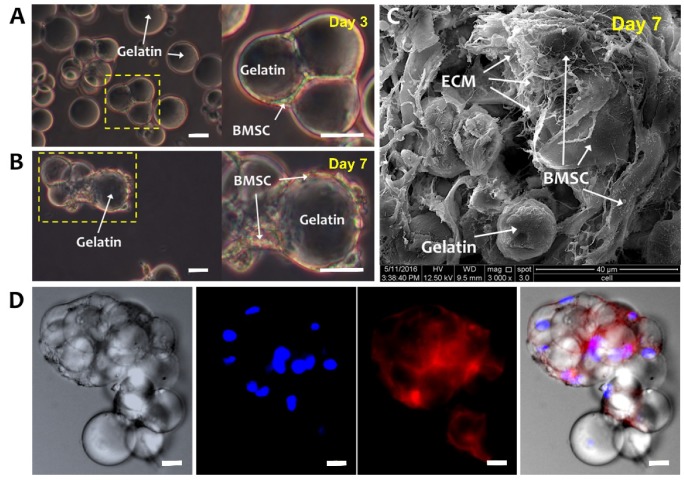
Bone-marrow-derived mesenchymal stem cells (BMSCs) cultured on GMs. Optical microscope images of BMSCs-GM on (**A**) Day 3 and (**B**) Day 7. White arrows were showing the bridging of adjacent GMs by elongated BMSCs, indicating cell–cell and cell–microsphere interactions. (**C**) SEM (3000× Magnification; scale bar: 40 µm) and (**D**) Confocal Laser Scanning Microscopy (CLSM) images of BMSCs-GM on Day 7. For the CLSM image, cell actin was stained with phalloidin-TRITC and nucleus with Hoechst. (**A**,**B**,**D**: 100× Magnification; scale bar: 100 µm).

Figure 2A,B are optical microscope images of BMSCs-GM on days 3 and 7. On day 3, BMSCs were well attached to GMs and demonstrated a flattened and elongated morphology. Moreover, GMs are shown to form aggregates, which enlarged at day 7 with extensive cell–cell and cell–matrix interactions (Figure 2B,C). Figure 2C illustrates the BMSCs and GMs under SEM, whereby the BMSCs secreted ECM surrounding the GMs. The fluorescent staining of the BMSCs-GM aggregates showed the formation of actin cytoskeleton, indicating the strong attachment and elongation of the BMSCs over GMs (Figure 2D).

### 2.3. Proliferation, Characterization, and Differentiation of BMSCs-TCP vs. BMSCs-GM

**Figure 3:**
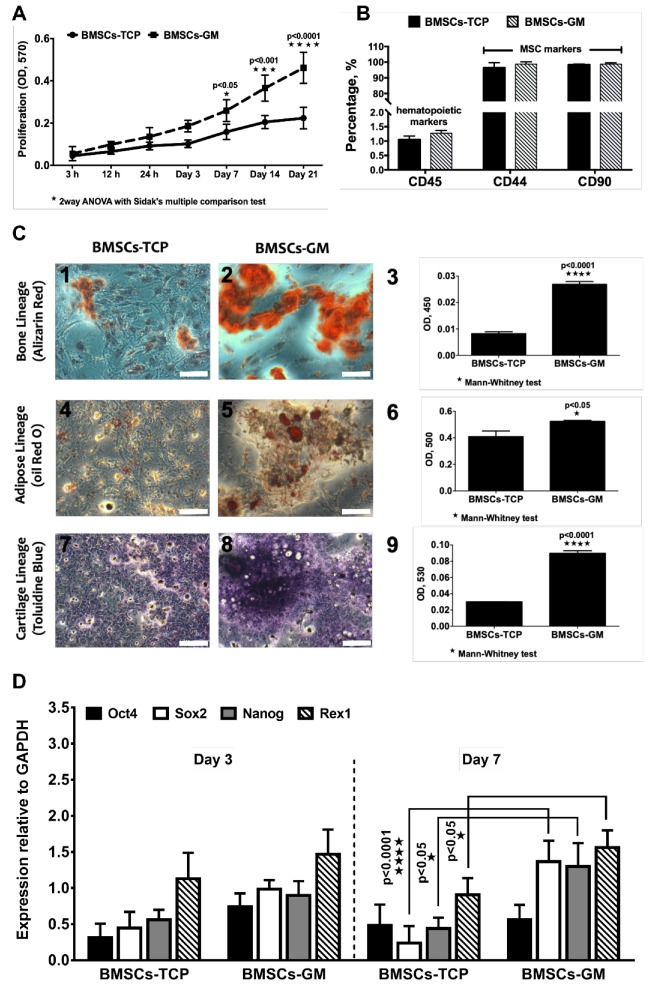
Cell growth, characterization and differentiation of BMSCs on a tissue culture plate (TCP) (2D) and GM (3D). (**A**) The proliferation of BMSCs-TCP and BMSCs-GM studied using Presto Blue assay. (**B**) Immunophenotype of BMSCs-TCP and BMSCs-GM via flow cytometry analysis (**C**) Optical microscope images of Alizarin Red for osteogenic differentiation, Oil Red O staining for adipogenic differentiation and Toluidine Blue staining for chondrogenic differentiation. (**D**) Stemness marker genes Oct4, Sox2, Nanog, and Rex1 of BMSCs-TCP and BMSCs-GM after day 3 and day 7. The sample size (*n*) for each experiment was six (*n* = 6). (BMSCs-TCP/day 3; BMSCs-GM/day 3; BMSCs-TCP/day 7; BMSCs-GM/day 7). (**C**: 100× Magnification; scale bar: 100 µm).

The growth and differentiation properties of BMSCs were evaluated in 2D and 3D culture systems, i.e., on a tissue culture plate (TCP) and GMs, respectively. Figure 3A shows the proliferation rate of BMSCs on GMs and TCP cultured in the FD medium, and, expectedly, the number of cells gradually increased with culture time until 21 days. When compared to TCP, the proliferation of BMSCs was significantly higher on GMs at day 7 (0.26 ± 0.10), day 14 (0.37 ± 0.16), and day 21 (0.46 ± 0.24). The flow cytometric analysis of surface markers demonstrated that >95% cells in both TCP and GM culture expressed MSC marker CD44 (Cat. No. 555478) and CD90 (Cat. No. 555595), and <5% of cells expressed hematopoietic marker CD45 (Cat. No. 555482) (Figure 3B). The stemness marker genes (Oct4, Nanog, Rex1, and Sox2) of BMSCs-GM and BMSCs-TCP were analyzed using qPCR (Figure 3D). There was no significant difference observed for those genes under both conditions on day 3. However, on day 7, three out of four genes (i.e., Sox2 = 1.36 ± 0.26, Nanog = 1.30 ± 0.20, and Rex1 = 1.60 ± 0.15) were significantly higher in BMSCs-GM compared to BMSCs-TCP. No significant difference recorded between day 3 and day 7 for BMSCs-GM as well as BMSCs-TCP. Moreover, BMSCs on TCP and GM can differentiate into three main cell lineages, namely osteocyte, adipocyte, and chondrocyte (Figure 3C). The absorbance values for BMSCs-GM were significantly higher compared to that of BMSCs-TCP (Alizarin Red = 0.026 ± 0.003, Oil Red O= 0.53 ± 0.02 and Toluidine Blue = 0.086 ± 0.01). For the tri-lineage differentiation experiment, data were not normalized to the cell number, hence we could not rule out the fact that the increased differentiation observed could also be due to higher number of cells in BMSC-GM. Nevertheless, the micrographs in Figure 3C show that the area and intensity of the staining was indeed higher in BMSC-GM.

### 2.4. Attachment and Proliferation of BMSCs-GM in a Static vs. Dynamic Culture Environment

**Figure 4:**
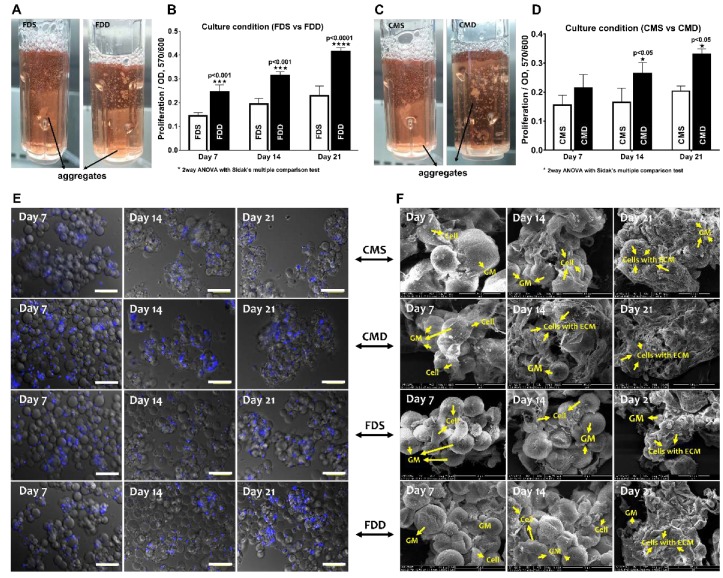
BMSCs attachment and proliferation in the static and dynamic culture system. Evaluation based on the different medium and culture system. (**A**,**B**) Macroscopically view and proliferation of BMSCs-GM in FDS and FDD culture condition (**C**,**D**) Macroscopic view and proliferation of BMSCs-GM in CMS and CMD culture condition. (**E**,**F**) Microscopic view of BMSCs-GM in FDD, FDS, CMD, and CMS. All experiments based on BMSCs-GM incubated in FD and chondrogenic induction medium (CIM) for 7, 14, and 21 days using static and dynamic culture condition (FD-Static (FDS), CIM-Static (CMS), FD-Dynamic (FDD), and CIM-Dynamic (CMD)). The sample size (*n*) for each experiment was six (*n* = 6). (**E**: 100× Magnification; scale bar: 100 µm **F**: 3000× Magnification; scale bar: 40 µm).

After confirming the superior growth and differentiation potential of BMSCs on GM under static culture condition, the effect of dynamic culture (BioLevitator^TM^) on BMSC properties were investigated compared to the static condition in two different mediums, namely FD and CIM. Gross appearance (Figure 4A,C) revealed that BMSCs-GM in the static culture system, i.e., static culture in FD (FDS) and static culture in CIM (CMS), exhibited larger and more aggregates compared to those in the dynamic condition (i.e., dynamic culture in FD (FDD) and dynamic culture in CIM (CMD). The proliferation of BMSCs-GM in all groups was analyzed using presto blue assay (Figure 4B,D). The proliferation of BMSCs was significantly higher in FDD at day 7 (0.24 ± 0.01), day 14 (0.31 ± 0.12), and day 21 (0.41 ± 0.20) compared to FDS. Meanwhile, BMSCs in CMD exhibited significantly higher proliferation at day 14 (0.26 ± 0.01) and day 21 (0.33 ± 0.13) compared to CMS. Microscopic observations (Figure 4E) have shown that BMSCs were still homogenously distributed on GMs in all groups at day 7, but aggregation can be observed later on day 14 and 21 in all groups. SEM images of BMSCs-GM under four different culture conditions (FDS, CMS, FDD, CMD) demonstrated the attachment and elongation of BMSCs with the production of extracellular matrix (ECM) (Figure 4F). The BMSCs deposited an abundant amount of ECM proteins that covered the surface of microspheres and demonstrated cell–cell and cell–microspheres binding in all groups. ECM protein can be seen more significantly in cultured BMSCs in CMD after 21 days of culture.

### 2.5. Chondrogenic Differentiation of BMSCs-GM in a Static vs. Dynamic Culture Environment

**Figure 5:**
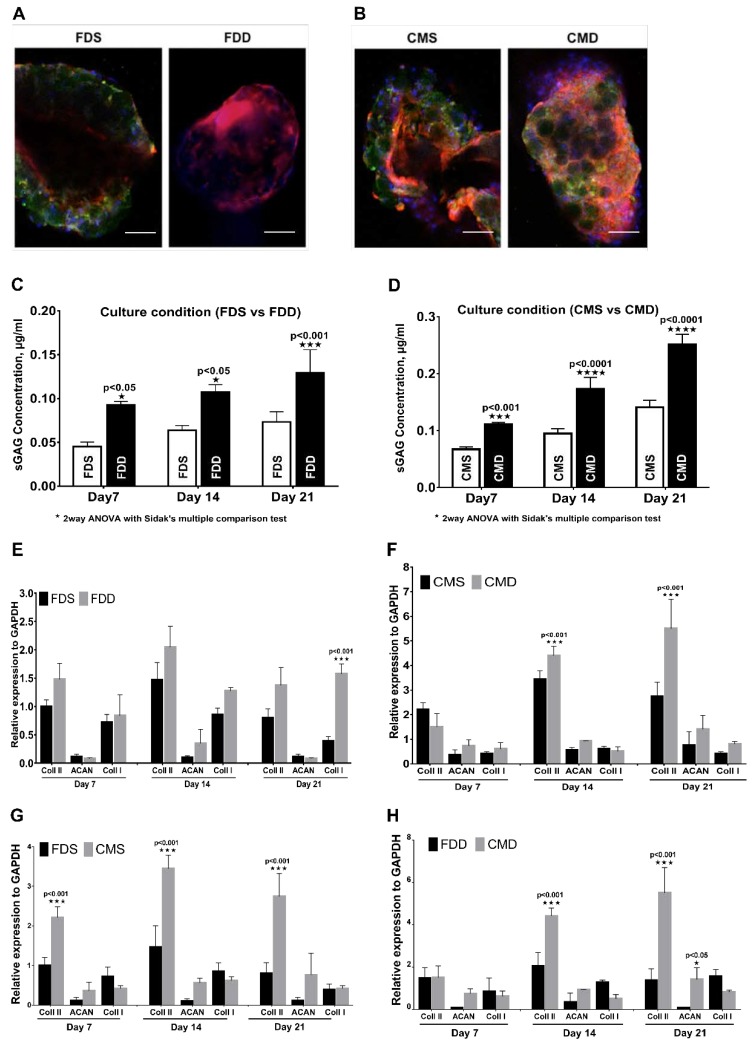
Chondrogenic differentiation of BMSCs in the static and dynamic cultural environment. Evaluation based on the different medium and culture system. (**A**,**B**) Immunohistochemistry staining with rabbit anti-human collagen II and counterstained with 4′,6-diamidino-2-phenylindole (DAPI) on day 21, (100× Magnification; scale bar: 100 µm). (**C**,**D**) Proteoglycan expression and (**E**–**H**) RNA expression of BMSCs on GM in FDD, FDS, CMD, and CMS. All experiments based on BMSCs-GM were incubated in FD and CIM for 7, 14, and 21 days using static and dynamic culture condition in FD and CIM mediums. Figure 4. BMSC attachment and proliferation in the static and dynamic culture system. Evaluation based on the different medium and culture system. (**A**,**B**) A macroscopic view and proliferation of BMSCs-GM in FDS and FDD culture condition. (**C**,**D**) A macroscopic view and proliferation of BMSCs-GM in CMS and CMD culture condition. (**E**,**F**) A macroscopic view of BMSCs-GM in FDD, FDS, CMD, and CMS. All experiments based on BMSCs-GM incubated in FD and CIM for 7, 14, and 21 days using static and dynamic culture condition (FD-Static (FDS), CIM-Static (CMS), FD-Dynamic (FDD), and CIM-Dynamic (CMD)). The sample size (*n*) for each experiment was six (*n* = 6). (Figure 4E: 100× magnification; scale bar: 100 µm. Figure 4F: 3000× Magnification; scale bar: 40 µm). (FD-Static (FDS), CIM-Static (CMS), FD-Dynamic (FDD), CIM-Dynamic (CMD)). (*p* ≤ 0.001: FDS vs. FDD, CMS vs. CMD, FDS vs. CMS and FDD vs. CMD).

The pictograph from immunohistochemical staining (Figure 5A,B) showed the expression of type II collagen in all culture conditions, whereby culturing in dynamic conditions in both media produced higher type II collagen compared with culturing in static condition. In terms of proteoglycan expression, cultured BMSCs in both FDD and CMD showed a significant increase in the expression of proteoglycan compared to those in FDS with increasing culturing time (Figure 5C,D). Figure 5E,F showed the chondrogenic gene expression analysis in different culture conditions. There were no significant changes in terms of type I collagen, aggrecan, and type II collagen expression between FDS and FDD except that the expression of type I collagen in FDD was significantly higher than in FDS on day 21. For CMS and CMD, the changes in mRNA expression were notable for type II collagen. As shown in Figure 5F, on days 14 and 21, the expression of type 2 collagen in the CMD was significantly higher than in the CMS. Figure 5G,H showed the chondrogenic gene expression analysis in different culture mediums within the same culture condition. There were significant changes (*p* < 0.001) of type II collagen expression between FDS and CMS with increasing culturing time (Figure 5G). Meanwhile, in Figure 5H, there were no significant changes in terms of type I collagen, aggrecan, and type II collagen expression between FDD and CMD on day 7. However, significant changes in type II collagen were seen on day 14 and 21, respectively.

## 3. Discussion

This study demonstrated the influence of 3D culture conditions, compared to the 2D monolayer culture, on the proliferation and chondrogenic differentiation of BMSCs. The study also showed the responsiveness of BMSCs in the 3D culture environment towards proliferation and chondrogenic differentiation when exposed to an external factor, such as mechanical agitation (dynamic culture). These data are in agreement with previous findings [34,35].

The work highlighted the proliferative and chondrogenic effects of GMs on BMSCs. Compared to the limited space in conventional 2D TCP, 3D GM provided a greater surface area for cell anchorage and growth [36]. The significant increase in the staining of BMSC-GM post lineage differentiation indicated the enhancement effect of GM on BMSC differentiation (Figure 3C1–9). However, the mechanism for such an effect of GM on BMSCs remains unclear, although some speculated that it could be due to the synergistic effect of increased proliferation of mesenchymal stem cells on GM [37]. Meanwhile, it was hinted that it could be the presence of amino acid sequences (arginine-glycine-aspartic acid (RGD) sequence) on gelatin, mimicking those in the native extracellular matrix of chondrocyte that further induces the differentiation of BMSCs [38].

On an interesting note, the study also revealed the upregulation of Sox2 in BMSCs-GM culture but not BMSCs-TCP on day 3. Subsequently, Rex1 and Nanog were upregulated in BMSCs-GM by day 7. Rex1 is known to be the key player in regulating human and mouse embryonic stem cell renewal and differentiation fate while Nanog, Sox2, and Oct4 play an integral role in regulating Rex1 [39,40]. Previous studies have reported the loss of stemness properties of stem cells in 2D monolayer culture due to the down-regulation of Oct4, Sox2, and Nanog [41]. This similar result observed in the BMSCs-TCP, whereby Nanog, Sox2, and Oct4 genes were down-regulated while the Rex1 gene was upregulated. In contrast, in BMSCs-GM, Rex1 was strongly expressed simultaneously with Sox2 and Nanog at day 7, indicating the maintenance of pluripotency in 3D culture even up to day 7 [42].

To ensure its regenerative capabilities, the maintenance of stemness capacities is paramount. One of the crucial parts in culturing BMSCs is to maintain their stemness properties during in vitro propagation [43]. The expression of specific CD markers and multilineage differentiation capabilities are indicators of the stemness properties of MSCs. In our study, the 3D BMSC-GM constructs maintained the BMSCs stemness properties by expressing the MSC markers, CD44 and CD90. The BMSCs cultured in both TCP and GM can be differentiated into mesodermal lineages, such as adipogenic, chondrogenic, and osteogenic, as reported in various 2D and 3D scaffolds [44]. Quantitatively, the staining of all three mesodermal lineages for BMSC-GM was higher than BMSC-TCP. Therefore, it could be safe to conclude that the 3D BMSC-GM culture is a well-suited technique to increase the expansion of cells without affecting the stemness properties of BMSCs. However, factors that are associated with the stemness maintenance on BMSCs still need to be elucidated [45].

In general, under static culture conditions, BMSC-GM increased the proliferation and chondrogenic differentiation of BMSCs compared to BMSC-TCP. However, static conditions have been proven to have limited seeding efficiencies due to the presence of concentration gradients and inefficient gas-liquid oxygen transfer [34]. Thus, dynamic culture was explored and has been widely applied in 3D cells-microcarriers culture [46,47]. The spinner flask system is the most applied technique for dynamic cell-microcarrier culture and is gently agitated in specific volumes of culture medium. These conditions potentially improved gas–liquid oxygen transfer, increase cell survival rates, and produce a high number of cells in culture [48].

In this study, BMSC-GM was applied to the dynamic culture conditions using Biolevitator^TM^ (Hamilton Company, Reno, NV, USA), an automated bioreactor. We first tested the effect of dynamic culture with FD medium (FDS vs. FDD) and later with CIM (CMS vs. CMD). The FD medium was prepared with standard fetal bovine serum, while the CIM was prepared with growth factors for chondrogenic induction. Thus, we could compare the proliferation and differentiation of BMSCs in both conditions, with or without a chondrogenic growth factor. In the FDS and CMS in static cultures, no significant growth in cell numbers was observed on days 7, 14, and 21. Even though gelatin is known to support cell proliferation, the number of cell yield in the static condition was still lower compared to FDD and CMD in a dynamic condition. Both groups in dynamic conditions showed higher cell numbers. The low cell growth on GMs, in static condition in our study was probably due to reduced seeding efficiency since the cells may plunge into the gaps between the microspheres. Seeded cells that attached to the GMs will survive, while those who dropped to the bottom of the non-adhesive tube will eventually die [26]. A different phenomenon in the case of a dynamic condition occurs whereby the repeating cycles of agitation and non-agitation will allow the cells to attach the the GM, as suggested by Perez et al. 2014, thus producing a high number of cells, particularly in the FDD. Our results concurs with that reported by Chang et al., whereby hydrophilic scaffold and hydrodynamic pressure exert a synergistic effect on cell growth [14].

Li et al. previously reported that tissue-engineered cartilage constructs cultured in the dynamic bioreactor resulted in increased tissue net weight gain and increased expression of chondrogenic genes, such as collagen types II and IX, cartilage oligomeric matrix protein, and aggrecan [49]. In this study, among the groups (FDS, CMS, FDD, CMD), CMD generally showed higher expression of type II collagen and proteoglycan significantly, with the highest expression recorded on Day 21. Although FDD exhibited a high number of proliferated cells, this condition produced a low expression of type II collagen. This result is probably due to the tendency of FDD to differentiate towards fibroblasts rather than chondrocytes.

One of the limitations with the conventional MSC intra-articular injection is the low number of MSCs that survived after injection, possibly due to the aggregation of cells upon delivery [50]. On the other hand, aggregates could hinder cell delivery as these would potentially clog the intra-articular needle. Therefore, aggregation should be controlled to avoid clump formation during the injection. The number of aggregations can be facilitated by regulating the mixing intensity, oxygen tension, and duration of culture [51,52]. The dynamic culture of 3D BMSC-GM serves as a potential technique to overcome this limitation by allowing continuous rotation of the 3D cell suspension in alternating directions [53]. The GM would provide a platform for the MSC to better proliferate and differentiate without affecting their quality, while the dynamic condition would reduce the clumping formation and hence improve the viability of the cells and prevent needle clogging.

An interesting observation from this study is that BMSC-GM culture produces significantly higher capabilities to differentiate adipocyte, chondrocyte, and osteocyte in comparison to BMSC-TCP. The question here is, how do we control the lineage differentiation to chondrocyte once we deliver the cells intraarticularly? The best option that we could learn from this study is that we could “prime” the BMSCs with CIM as the data from chondrogenic gene expression indicated that CIM would guide the BMSC to the chondrocyte lineage. Together, the combination of dynamic, CIM, and GM culture would provide an ideal BMSC expansion technique for cell delivery.

While the technique seemed promising, we also pondered upon how this innovation will be applicable for clinical intervention. Undeniably, the process of obtaining the final product to be used clinically is time consuming, from sample processing, in vitro cell expansion, and priming. Hence, we believe that the usage of this technology will be applicable for cartilage diseases, such as late stage osteoarthritis, that are resistant to any form of conventional therapy and require elective surgical procedures. The cost of this potential therapy is predicted to be high due to the fact that it is a form of tailored and personalized therapy with stringent GMP requirements. Mutual understanding about the potential benefits of this therapy and the cost between healthcare providers and patients must be addressed. This minimally invasive procedure therapy may potentially outweigh its cost if it results in permanent cartilage regeneration and the reduction of pain for a significant period of time.

## 4. Materials and Methods

### 4.1. Isolation of BMSCs and Cell Culture

Bone marrow samples were collected from six patients (*n* = 6) undergoing a total knee replacement procedure with their prior written consents. Five to ten milliliters of bone marrow aspirates were obtained from the knee joints of the patients. The collected BMSCs were isolated and cultured in a tissue culture plate (TCP) according to the method previously described [19]. Briefly, the samples were processed within 6–12 h after collection. BMSCs were isolated using the Ficoll-Paque method. The BMSCs were then cultured in FD [F12: DMEM (1:1) supplemented with 10% fetal bovine serum (FBS; Biowest, Riverside, MO, USA), 1% antibiotic-antimycotic (Gibco, Grand Island, NY, USA), 1% glutamax (Gibco, Grand Island, NY, USA), and 1% vitamin C (Sigma-Aldrich, St. Louis, MO, USA]. The cells were incubated at 37 °C in a humidified atmosphere with 5% CO_2_. The culture medium was changed every 2–3 days until it reaches 70%–80% confluence. Cells were trypsinized using 0.05% Trypsin–EDTA (Gibco, Grand Island, NY, USA) and subcultured until passage 3. Passage 3 cells were used for subsequent experiments.

### 4.2. Preparation and Characterization of Gelatin Microspheres (GM)

GM were fabricated using a water-in-oil emulsion method, as described previously [29]. A solution (20 mL) of 10 wt% gelatin (the isoelectric point = 5.0, the weight-average molecular weight = 100,000, Nitta Gelatin Inc., Osaka, Japan) was preheated at 40 °C, and then added dropwise into 600 mL of olive oil (Wako, Ltd., Osaka, Japan) at 40 °C, followed by stirring at 400 rpm for 10 min to prepare a water-in-oil emulsion. The synthesized microspheres were washed three times with cold acetone by combination with centrifugation (5000 rpm, 4 °C, and 5 min) to exclude residual oil completely. Then, they were fractionated in size by sieves with apertures of 20, 32, and 53 µm (Endecotts, Ltd., London, UK) and air-dried at 4 °C. The non-crosslinked and dried GM (200 mg) were treated in a vacuum oven at 140 °C and 0.1 torr for the dehydrothermal crosslinking of gelatin according to the method as previously reported [17].

GMs were characterized for their size using an optical microscope and its surface morphology by a scanning electron microscope (SEM). The size of microspheres was measured both in dry and in a wet state after immersion in sterile deionized water for 3 h. Images were captured using a camera attached to the optical microscope, and the size was analyzed by measuring the diameters of the microspheres using image Q-Capture Pro 7 (version 7.0.5). For the imaging with SEM, GM with or without cells were mounted onto aluminum stubs using black carbon tapes and sputter-coated with gold (Sputter Coater Q150TES, Quorum, Italy). The specimen surface was examined using high-resolution SEM (FEI, Quanta 200, UK) at a pressure of 0.1 m Torr at an accelerating voltage of 15 kV.

### 4.3. Cell Seeding on Gelatin Microspheres and Tissue Culture Plate (TCP)

GM were sterilized with 70% ethanol, followed by complete washing with sterilized phosphate buffer saline (PBS; Sigma-Aldrich, St. Louis, MO, USA). Before transferring the GM onto the TCP, the plates have to be coated with polyvinyl alcohol (PVA; Sigma-Aldrich, St. Louis, MO, USA, polymerization degree of 1800 and 88 mole % saponification) in order to prevent cell attachment onto the plate. Briefly, 1% PVA in PBS was added into each well of 12- and 24-well (1mL/well) and incubated at 37 °C for 15 min. Then, the solution was removed by aspiration, and the well was washed with PBS (1mL/well) twice. For cell differentiation experiments, GM were transferred to 12-well plates at 10 mg per well, and 5 × 10^4^ BMSCs at passage 3 were seeded onto the microspheres per well (i.e., 5 × 10^4^ cells per mg of microspheres). For cell proliferation experiments, GMs were transferred to 24-well plates at 2mg per well, and 1 × 10^4^ cells per well were subsequently seeded onto the microsphere. The same number of cells were cultured on non-PVA-coated TCP as control, which was later compared to the microsphere group in both experiments.

### 4.4. Immunophenotype Analysis of BMSCs-TCP vs. BMSCs-GM by Flow Cytometry

Cells in BMSCs-TCP and BMSCs-GM culture were isolated with 0.05% trypsin-EDTA, washed with 0.2% bovine serum albumin (BSA) in PBS, and stained with the following list of FITC conjugated mouse anti-human antibodies: CD44 (Cat. No. 555478), CD45 (Cat. No. 555482), and CD90 (Cat. No. 555595) (BD Pharmingen, San Jose, CA, USA). In brief, 2 × 10^5^ cells were suspended in 100 µL of 0.2% BSA in PBS and stained with individual antibodies (at a concentration as per manufacturer’s recommendation) in separate tubes for 30 min. The cells were then washed with 0.2% BSA/PBS twice and fixed in 4% paraformaldehyde (Sigma-Aldrich, St. Louis, MO, USA). The samples were washed twice in PBS, suspended in 0.2% BSA/PBS, and analyzed by FACS Calibur^TM^ flow cytometer system (BD Biosciences, San Jose, CA, USA) using CellQuest Pro software (version 5.1). Ten thousand gated events were recorded. Gating was determined using unstained cells as a negative control.

### 4.5. Real-Time Polymerase Chain Reaction (qPCR)

Total RNA from BMSC cultures (BMSCs-TCP and BMSCs-GM) were extracted using the Qiagen RNeasy mini kit. The concentrations of the extracted total RNA were determined using Nanodrop 2000 spectrophotometer (Thermo Fisher Scientific, Waltham, MA, USA). Complementary DNA (cDNA) was synthesized from the extracted RNA using Maxima first strand cDNA synthesis kit (Thermo Fisher Scientific, USA). The obtained cDNA was used for qPCR, which was performed in triplicates by using the SYBR FAST Biorad qPCR master mix (Bio-rad, Hercules, CA, USA). The primers used in the qPCR are given in Table 1. The housekeeping gene, GAPDH, was used for gene expression data normalization. The experiment was performed in triplicates.

### 4.6. Proliferation Assay on BMSCs-TCP vs. BMSCs-GM

Presto Blue (Thermo Fisher Scientific, Waltham, MA, USA) was used to study the viability of BMSCs cultured on GMs (BMSCs-GM) and TCP (BMSCs-TCP). These two groups were cultured in FD for 21 days in replicates, and the data were collected at different time points for analysis (3, 12, and 24 h, day 3, 7, 14, and 21). All the respective culture and blank plates were rinsed with PBS, and then 180 µl of FBS-free medium was added into each plate followed by 20 µL of Presto Blue reagent on top of the FBS-free medium. Later, the plates were incubated for 2 h at 37 °C and 5% CO2. After 2 h of incubation, 100 µL of the supernatant was transferred into a 96-well plate, and, subsequently, absorbance was measured using a microplate reader at 570 nm against a reference wavelength of 600 nm. The experiment was performed in triplicates.

### 4.7. Trilineage Differentiation of BMSCs-TCP vs. BMSCs-GM

The BMSCs-TCP and BMSCs-GM were cultured in three different culture media for multilineage differentiation. Osteogenic differentiation in both groups was prolonged until 21 days in the FD medium (Gibco, Grand Island, NY, USA) supplemented with 0.1mM dexamethasone (Sigma–Aldrich, St. Louis, MO, USA), 10mM b-glycerol phosphate (Sigma–Aldrich, St. Louis, MO, USA), and 0.2mM ascorbic acid-2-phosphate (Sigma–Aldrich, St. Louis, MO, USA). Induced BMSCs were then replenished with fresh medium every 3 days. Differentiation activity was assessed with Alizarin Red staining, which positively stained for calcium deposition. Briefly, samples were fixed with cold ethanol for 1 h, rinsed with PBS, and stained with Alizarin Red for 1 h. The excess stain was washed off using PBS, followed by incubation with boric acid buffer and counterstained with hematoxylin. The samples were dried and evaluated using a bright field microscope (Olympus-CK40, Tokyo Japan). Alizarin Red dye was dissolved using a solution of 20% methanol and 10% acetic acid in water. After 15 min, the liquid was transferred to a 96-well plate and read on the spectrophotometer at 450 nm.

For adipogenic differentiation, BMSCs were cultured in FD medium (Gibco, Grand Island, NY, USA ) supplemented with 0.25 mmol/L 3-isobutyl-1-methylxanthine (Sigma–Aldrich, St. Louis, MO, USA), 100 nmol/mL dexamethasone, and 100 nmol/L human recombinant insulin (Sigma–Aldrich, St. Louis, MO, USA). The medium was changed every 3 days. After day 21, samples were stained with Oil Red O to identify lipid deposition. Adipogenic cultures were rinsed once with PBS and fixed with 10% formalin for 60 min at room temperature. The formalin was discarded, and the samples were stained with 0.36% Oil Red O for 50 min. The samples were subsequently examined using a bright field microscope. Oil Red O dye was dissolved by adding 100% isopropanol and incubation for 10 min. The liquid was measured on a spectrophotometer at 500 nm.

For chondrogenic differentiation, BMSCs were cultured in chondrogenic induction medium (CIM), which comprise of FD medium (Gibco, Grand Island, NY, USA) supplemented with serum, 1% Insulin Transferring Selenium (ITS) (Gibco, Grand Island, NY, USA), 0.2mM ascorbic acid-2 phosphate (Sigma, St. Louis, MO, USA), 40 ng/mL L-proline (Sigma, St. Louis, MO, USA), 100 nM dexamethasone (Invitrogen Inc., Waltham, MA, USA), 10 ng/mL transforming growth factor beta 3 (TGF-β3) (Invitrogen Inc., Waltham, MA, USA), and 50 ng/mL insulin-like growth factor 1(IGF-1) (Invitrogen Inc., Waltham, MA, USA) [54,55]. The medium was changed every 3 days, and the induction period lasted for 21 days. After 21 days, the samples were fixed and stained with toluidine blue to identify the presence of proteoglycans by using a bright field microscope. Toluidine Blue dye was dissolved using a solution consisted of 2.5 mL of concentrated sulfuric acid and 2.5 mL of water in 95 mL of methanol. The liquid was then transferred to a 96-well plate on the spectrophotometer and measured at 635 nm.

### 4.8. Chondrogenic Differentiation of BMSCs-GM in a Static vs. Dynamic Culture Environment

**Figure 6:**
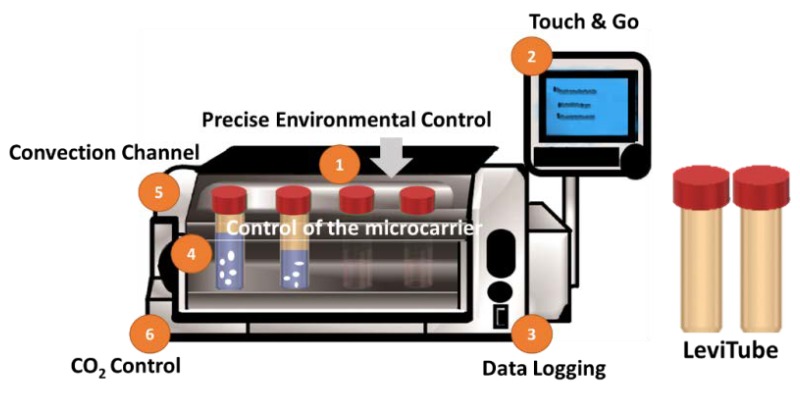
The bench-top incubator BioLevitator^TM^ which provides 3D static and dynamic culture system in 50 mL centrifuge tubes (LeviTubes™).

In order to simulate a dynamic culture environment, continuous mechanical agitation of the culture media was performed using the BioLevitator^TM^ machine (Hamilton Company, Reno, NV, USA) (Figure 6). Precise digital control of the agitation parameters during inoculation was applied. The applied setting was 3 sec of rotation period, 0 sec of rotation pause, and 75 rpm of rotation speed. Followed by a 2-min agitation period and 45-min agitation pause for intermittent inoculation. The total efficient loading of the GM was 24 h. LeviTubes™ (Global Cell Solutions), which are 50-mL suspension culture tubes that have small baffles on the inner walls which keep cells or microcarriers suspended in media by simple oscillation of the LeviTubes™.

Two different media, F12: DMEM (1:1) + 10% FBS (FD) and chondrogenic induction medium (CIM), were used in this experiment. A week before the experiment, a total of 2.5 × 10^5^ cells were inoculated onto 50 mg of gelatin microsphere in 50 mL of FD medium in a LeviTube™ loaded onto the BioLevitator^TM^ machine. The medium was then replaced with 50 mL of CIM and was subjected to either static or dynamic conditions (oscillation of the LeviTubes™ at a rotation speed at 75 rpm) and was initiated within 10 s after loading into the machine and continuously rotated till the end of the process) condition. BMSCs suspended in FD medium in either static or dynamic conditions acted as the control for their corresponding CIM group, respectively. The samples were evaluated for chondrogenic markers using qPCR, proteoglycan expression using kits (Biocolor, Belfast, UK) at 7, 14, and 21 days, and immunofluorescent staining at day 21.

### 4.9. Immunofluorescence Staining

The samples were washed with Dulbecco’s PBS and fixed with 4% paraformaldehyde for overnight, permeabilized for 20 min with 0.5% Triton X-100 solution (Sigma-Aldrich, St. Louis, MO, USA), and then blocked with 10% goat serum for 1 h at 37 °C. The cells were incubated with Mouse Anti-Human Collagen II (Abcam, Cambridge, MA, USA) for chondrocytes overnight at 4 °C. The cells were incubated with Alexa Fluor 594 goat anti-mouse antibodies (Invitrogen Inc., Waltham, MA, USA) on the following day for 1 h at 37 °C and counterstained with DAPI (Dako, Glostrup Denmark) for 15 min. The cells were observed and evaluated using the Nikon Eclipse Ti fluorescence microscope (Nikon, Japan).

### 4.10. Sulfated Glycosaminoglycan (sGAG) Production Assay

All samples (BMSCs-GM and BMSCs-TCP) were digested with a papain digestion solution (125 μg/mL papain, 5 mM L-cystein, 100 mM Na2HPO4 and 5 mM EDTA; pH 6.8) at 60 °C for 16 h. Sulfated glycosaminoglycan content was analyzed using a 1,9-dimethyl methylene blue (DMMB) assay (Biocolor, Belfast, UK). A 20 μL aliquot of each sample was pipetted into the microplate reader and added with 200 μL DMMB. Samples were analyzed immediately by measuring the absorbance at 525 nm [56]. The experiment was run in triplicates.

### 4.11. Statistical Analysis

The results were expressed as the mean ± standard error of the mean (SEM). Statistical analysis was performed using using GraphPad Prism 7.0. For comparison between two groups, a Mann–Whitney test was used. For comparison across three or more groups, all data sets have passed the normality test, including the D’Agostino-Pearson omnibus and Shapiro–Wilk normality tests before analyzing using two-way ANOVA and Sidak’s multiple comparison tests. The differences were considered significant when *p* < 0.05.

## 5. Conclusions

This study reveals that the dynamic culture condition could be beneficial for cartilage tissue engineering cultures. This condition could reinvent the current conventional 2D or 3D of stem cells. This approach also enhanced cell–cell and cell–matrix interactions, preserved the stemness properties, and enhanced the differentiation abilities of BMSCs into adipogenic, osteogenic, and chondrogenic lineages when compared to the traditional 2D cultures. Cell attachment and proliferation on the GMs were substantially increased in the dynamic culture condition compared to in the static condition.

Furthermore, we found that FD media is potentially suitable for cell proliferation but not for differentiation. CIM media were found as the right candidate for the differentiation of BMSCs-GM that produces higher type II collagen. In light of the experiments discussed, GM is beneficial as a suitable cell delivery agent for cartilage repair or regeneration. However, more studies are required to address specific issues with the GM, particularly in terms of its role in chondrogenic differentiation and also cellular homing.

## Figures and Tables

**Table 1:** Primer sequences used for real-time PCR experiments.

Name	Forward 5′-3′	Reverse 5′-3′
Type 1 Col	AAGGCTTCAAGGTCCCCCTGGTG	CAGCACCAGTAGCACCATCATTTC
Type 2 Col	GGCAATAGCAGGTTCACGTACA	CGATAACAGTCTTGCCCCACTT
Aggrecan	ACTTCCGCTGGTCAGATGGA	TCTCGTGCCAGATCATCACC
GAPDH	GGCGATGCTGGCGCTGAGTAC	TGGTTCACACCCATGACGA
Oct4	GCAGCGACTATGCACAACGA	CCAGAGTGGTGACGGAGACA
Sox2	CATCACCCACAGCAAATGACA	GCTCCTACCGTACCACTAGAACTT
Nanog	CCTGTGATTTGTGGGCCTG	GACAGTCTCCGTGTGAGGCAT
Rex1	TGAAAGCCCACATCCTAACG	TATAACCGCTTTTGGGGTTG

## Abbreviations

3D	Three-Dimensional
2D	Two-Dimensional
BMSCs	Bone-Marrow-Derived Mesenchymal Stem Cells
GMs	Gelatin Microspheres
MSC	Mesenchymal Stem Cells
FBS	Fetal Bovine Serum
SEM	Scanning Electron Microscope
TCP	Tissue Culture Plate
PVA	Polyvinyl Alcohol
PBS	Phosphate Buffer Saline
BSA	Bovine Serum Albumin
CIM	Chondrogenic Induction Medium
ITS	Insulin Transferring Selenium
TGF-β3	Transforming Growth Factor Beta 3
IGF-1	Insulin-Like Growth Factor 1
DMMB	1,9-Dimethyl Methylene Blue
CLSM	Confocal Laser Scanning Microscopy
DAPI	4′,6-diamidino-2-phenylindole

## References

[1] Dzobo K., Thomford N.E., Senthebane D.A., Shipanga H., Rowe A., Dandara C., Pillay M., Shirley K., Motaung C.M. (2018). Innovation and Transformation of Medicine. Stem Cells Int..

[2] Gómez-Leduc T., Hervieu M., Legendre F., Bouyoucef M., Gruchy N., Poulain L., De Vienne C., Herlicoviez M., Demoor M., Galéra P. (2016). Chondrogenic commitment of human umbilical cord blood-derived mesenchymal stem cells in collagen matrices for cartilage engineering. Sci. Rep..

[3] Arslan E., Guler M.O., Tekinay A.B. (2016). Glycosaminoglycan-Mimetic Signals Direct the Osteo/Chondrogenic Differentiation of Mesenchymal Stem Cells in a Three-Dimensional Peptide Nanofiber Extracellular Matrix Mimetic Environment. Biomacromolecules.

[4] Fong C.Y., Subramanian A., Gauthaman K., Venugopal J., Biswas A., Ramakrishna S., Bongso A. (2012). Human Umbilical Cord Wharton’s Jelly Stem Cells Undergo Enhanced Chondrogenic Differentiation when Grown on Nanofibrous Scaffolds and in a Sequential Two-stage Culture Medium Environment. Stem Cell Rev. Reports.

[5] Negoro T., Takagaki Y., Okura H., Matsuyama A. (2018). Trends in clinical trials for articular cartilage repair by cell therapy. NPJ Regen. Med..

[6] Najar M., Bouhtit F., Melki R., Afif H., Hamal A., Fahmi H., Merimi M., Lagneaux L. (2019). Mesenchymal Stromal Cell-Based Therapy: New Perspectives and Challenges. J. Clin. Med..

[7] Wang L.T., Ting C.H., Yen M.L., Liu K.J., Sytwu H.K., Wu K.K., Yen B.L. (2016). Human mesenchymal stem cells (MSCs) for treatment towards immune- and inflammation-mediated diseases: Review of current clinical trials. J. Biomed. Sci..

[8] Emadedin M., Liastani M.G., Fazeli R., Mohseni F., Moghadasali R., Mardpour S., Hosseini S.E., Niknejadi M., Moeininia F., Fanni A.A. (2015). Long-term follow-up of intra-articular injection of autologous mesenchymal stem cells in patients with knee, ankle, or hip osteoarthritis. Arch. Iran. Med..

[9] Jo C.H., Chai J.W., Jeong E.C., Oh S., Shin J.S., Shim H., Yoon K.S. (2017). Intra-articular Injection of Mesenchymal Stem Cells for the Treatment of Osteoarthritis of the Knee: A 2-Year Follow-up Study. Am. J. Sports Med..

[10] Orozco L., Munar A., Soler R., Alberca M., Soler F., Huguet M., Sentís J., Sánchez A., García-Sancho J. (2013). Treatment of knee osteoarthritis with autologous mesenchymal stem cells: A pilot study. Transplantation.

[11] Banfi A., Muraglia A., Dozin B. (2000). Proliferation kinetics and differentiation potential of ex vivo expanded human bone marrow stromal cells: Implications for their use in cell therapy. Exp. Hematol..

[12] Justice B.A., Badr N.A., Felder R.A. (2009). 3D cell culture opens new dimensions in cell-based assays. Drug Discov. Today.

[13] Reiser J., Zhang X.Y., Hemenway C.S., Mondal D., Pradhan L., La Russa V.F. (2005). Potential of mesenchymal stem cells in gene therapy approaches for inherited and acquired diseases. Expert Opin. Biol. Ther..

[14] Chang N.J., Jhung Y.R., Issariyakul N., Yao C.K., Yeh M.L. (2012). Synergistic stimuli by hydrodynamic pressure and hydrophilic coating on PLGA scaffolds for extracellular matrix synthesis of engineered cartilage. J. Biomater. Sci. Polym. Ed..

[15] Cukierman E., Pankov R., Stevens D.R., Yamada K.M. (2016). Taking Cell-Matrix Adhesions to the Third Dimension. Science.

[16] Nakaguchi K., Jinnou H., Kaneko N., Sawada M., Hikita T., Saitoh S., Tabata Y., Sawamoto K. (2012). Growth factors released from gelatin hydrogel microspheres increase new neurons in the adult mouse brain. Stem Cells Int..

[17] Ozeki M., Tabata Y. (2005). In vivo degradability of hydrogels prepared from different gelatins by various cross-linking methods. J. Biomater. Sci. Polym. Ed..

[18] Shamsul B.S., Tan K.K., Chen H.C., Aminuddin B.S., Ruszymah B.H.I. (2014). Posterolateral spinal fusion with ostegenesis induced BMSC seeded TCP/HA in a sheep model. Tissue Cell.

[19] Sulaiman S.B., Keong T.K., Cheng C.H., Saim A.B., Idrus R.B.H. (2013). Tricalcium phosphate/hydroxyapatite (TCP-HA) bone scaffold as potential candidate for the formation of tissue engineered bone. Indian J. Med. Res..

[20] Csd L., Am N., Ea W., Oa B., Bd B., Schwartz Z. (2014). Adipose Stem Cell Microbeads as Production Sources for Chondrogenic Growth Factors. J. Stem Cells Regen. Med..

[21] Meyer C., Stenberg L., Gonzalez-Perez F., Wrobel S., Ronchi G., Udina E., Suganuma S., Geuna S., Navarro X., Dahlin L.B. (2016). Chitosan-film enhanced chitosan nerve guides for long-distance regeneration of peripheral nerves. Biomaterials.

[22] Yuan Y., Kallos M.S., Christopher H., Arindom S. (2012). Improved expansion of human bone marrow-derived mesenchymal stem cells in microcarrier-based suspension culture. J. Tissue Eng. Regen. Med..

[23] Tharmalingam T., Sunley K., Spearman M., Butler M. (2011). Enhanced production of human recombinant proteins from CHO cells grown to high densities in macroporous microcarriers. Mol. Biotechnol..

[24] Leong W., Wang D.A. (2015). Cell-laden Polymeric Microspheres for Biomedical Applications. Trends Biotechnol..

[25] Tsai A.C., Ma T. (2016). Expansion of Human Mesenchymal Stem Cells in a Microcarrier Bioreactor. Methods Mol. Biol..

[26] Merten O.W. (2015). Advances in cell culture: Anchorage dependence. Philos. Trans. R. Soc. B Biol. Sci..

[27] Malda J., Frondoza C.G. (2006). Microcarriers in the engineering of cartilage and bone. Trends Biotechnol..

[28] Leong W., Lau T.T., Wang D.A. (2013). A temperature-cured dissolvable gelatin microsphere-based cell carrier for chondrocyte delivery in a hydrogel scaffolding system. Acta Biomater..

[29] Hayashi K., Tabata Y. (2011). Preparation of stem cell aggregates with gelatin microspheres to enhance biological functions. Acta Biomater..

[30] Ogawa T., Akazawa T., Tabata Y. (2010). In vitro proliferation and chondrogenic differentiation of rat bone marrow stem cells cultured with gelatin hydrogel microspheres for TGF-β1 release. J. Biomater. Sci. Polym. Ed..

[31] Gohi B.F.C.A., Liu X.Y., Zeng H.Y., Xu S., Ake K.M.H., Cao X.J., Zou K.M., Namulondo S. (2020). Enhanced efficiency in isolation and expansion of hAMSCs via dual enzyme digestion and micro-carrier. Cell Biosci..

[32] Guo T., Yu L., Lim C.G., Goodley A.S., Xiao X., Placone J.K., Ferlin K.M., Nguyen B.N.B., Hsieh A.H., Fisher J.P. (2016). Effect of Dynamic Culture and Periodic Compression on Human Mesenchymal Stem Cell Proliferation and Chondrogenesis. Ann. Biomed. Eng..

[33] Aggarwal A., Mehta S., Gupta D., Sheikh S., Pallagatti S., Singh R., Singla I. (2012). Clinical & immunological erythematosus patients characteristics in systemic lupus Maryam. J. Dent. Educ..

[34] Perez R.A., Riccardi K., Altankov G., Ginebra M.P. (2014). Dynamic cell culture on calcium phosphate microcarriers for bone tissue engineering applications. J. Tissue Eng..

[35] Agrawal P., Pramanik K., Biswas A., Ku Patra R. (2018). In vitro cartilage construct generation from silk fibroin- chitosan porous scaffold and umbilical cord blood derived human mesenchymal stem cells in dynamic culture condition. J. Biomed. Mater. Res. Part A.

[36] Schop D., Janssen F.W., Borgart E., de Bruijn J.D., van Dijkhuizen-Radersma R. (2008). Expansion of mesenchymal stem cells using a microcarrier-based cultivation system: Growth and metabolism. J. Tissue Eng. Regen. Med..

[37] Dexheimer V., Frank S., Richter W. (2012). Proliferation as a requirement for in vitro chondrogenesis of human mesenchymal stem cells. Stem Cells Dev..

[38] Ratner B.D., Hoffman A.S., Schoen F.J., Lemons J.E. (2013). Biomaterials Science: An Introduction to Materials in Medicine.

[39] Lee K.C., Wong W.K., Feng B. (2013). Decoding the pluripotency network: The emergence of new transcription factors. Biomedicines.

[40] Shi W., Wang H., Pan G., Geng Y., Guo Y., Pei D. (2006). Regulation of the Pluripotency Marker Rex-1 by Nanog and Sox2. J. Biol. Chem..

[41] Cheng N.C., Wang S., Young T.H. (2012). The influence of spheroid formation of human adipose-derived stem cells on chitosan films on stemness and differentiation capabilities. Biomaterials.

[42] Bhandari D.R., Seo K.-W., Roh K.-H., Jung J.-W., Kang S.-K., Kang K.-S. (2010). REX-1 expression and p38 MAPK activation status can determine proliferation/differentiation fates in human mesenchymal stem cells. PLoS ONE.

[43] Drela K., Stanaszek L., Nowakowski A., Kuczynska Z., Lukomska B. (2019). Experimental strategies of mesenchymal stem cell propagation: Adverse events and potential risk of functional changes. Stem Cells Int..

[44] Li W.J., Tuli R., Huang X., Laquerriere P., Tuan R.S. (2005). Multilineage differentiation of human mesenchymal stem cells in a three-dimensional nanofibrous scaffold. Biomaterials.

[45] Leong D.T., Abraham M.C., Gupta A., Lim T.-C., Chew F.T., Hutmacher D.W. (2012). ATF5, a possible regulator of osteogenic differentiation in human adipose-derived stem cells. J. Cell. Biochem..

[46] Boo L., Selvaratnam L., Tai C. (2011). Expansion and preservation of multipotentiality of rabbit bone-marrow derived mesenchymal stem cells in dextran-based microcarrier spin culture. J. Mater. Sci. Mater. Med..

[47] Yang Y., Rossi F.M., Putnins E.E. (2007). Ex vivo expansion of rat bone marrow mesenchymal stromal cells on microcarrier beads in spin culture. Biomaterials.

[48] Freed L.E., Vunjak-Novakovic G., Langer R. (1993). Cultivation of cell-polymer cartilage implants in bioreactors. J. Cell. Biochem..

[49] Li W.J., Jiang Y.J., Tuan R.S. (2008). Cell-nanofiber-based cartilage tissue engineering using improved cell seeding, growth factor, and bioreactor technologies. Tissue Eng. Part. A.

[50] Shim G., Lee S., Han J., Kim G., Jin H., Miao W., Yi T.G., Cho Y.K., Song S.U., Oh Y.K. (2015). Pharmacokinetics and in Vivo Fate of Intra-Articularly Transplanted Human Bone Marrow-Derived Clonal Mesenchymal Stem Cells. Stem Cells Dev..

[51] Malda J., van den Brink P., Meeuwse P., Grojec M., Martens D.E., Tramper J., Riesle J., van Blitterswijk C.A. (2004). Effect of oxygen tension on adult articular chondrocytes in microcarrier bioreactor culture. Tissue Eng..

[52] Frondoza C., Sohrabi A., Hungerford D. (1996). Human chondrocytes proliferate and produce matrix components in microcarrier suspension culture. Biomaterials.

[53] Fischer B., Meier A., Dehne A., Salhotra A., Tran T.A., Neumann S., Schmidt K., Meiser I., Neubauer J.C., Zimmermann H. (2018). A complete workflow for the differentiation and the dissociation of hiPSC-derived cardiospheres. Stem Cell Res..

[54] Ude C.C., Seet W.T., Aini S.S., Aminuddin B.S., Ruszymah B.H.I. (2018). Shelf Life Evaluation of Clinical Grade Chondrogenic Induced Aged Adult Stem Cells for Cartilage Regeneration. Sci. Rep..

[55] Ude C.C., Sulaiman S.B., Min-Hwei N., Hui-Cheng C., Ahmad J., Yahaya N.M., Saim A.B., Idrus R.B.H. (2014). Cartilage regeneration by chondrogenic induced adult stem cells in osteoarthritic sheep model. PLoS ONE.

[56] Shamsul B.S., Chowdhury S.R., Norhamdan M.Y., Ruszymah B.H.I. (2019). Effect of cell density on formation of three-dimensional cartilaginous constructs using fibrin & human osteoarthritic chondrocytes. Indian J. Med. Res..

